# Development and Evaluation of a Three-Dimensional-Printed Pediatric Intraosseous Infusion Simulator To Enhance Medical Training

**DOI:** 10.7759/cureus.21080

**Published:** 2022-01-10

**Authors:** Ryan E Wade, Brent McCullum, Chris Patey, Adam Dubrowski

**Affiliations:** 1 Faculty of Medicine, Memorial University of Newfoundland, St. John’s, CAN; 2 Emergency Department, Dalhousie Medicine New Brunswick, Moncton, CAN; 3 Emergency Medicine, Carbonear General Hospital, Carbonear, CAN; 4 Research and Development, Ontario Tech University, Oshawa, CAN

**Keywords:** three-dimensional (3d) printing, trauma pediatric, intraosseous infusion, simulation in medical education, emergency medicine and trauma

## Abstract

Vascular access is an essential and rate-limiting step during pediatric resuscitation efforts. Intraosseous (IO) access, an effective resuscitative strategy, remains underutilized in emergency departments. Many medical graduates report never performing the procedure before graduation, and it has been recommended that continuing education and in-servicing programs be implemented to increase the use and familiarity of IO access. The goal of this technical report is to describe the development and evaluation of a three-dimensional (3D)-printed Pediatric IO Infusion Model for simulation-based medical education. The simulator was designed by combining open-source models of a human skeleton and a lower leg surface scan in Blender (Blender Foundation, Amsterdam, Netherlands; www.blender.org), scaled to a pediatric size, and manipulated further using a JavaScript program. Polylactic acid was used to simulate bone while silicone molds were used as skin and soft tissue. Two trainers were produced and evaluated by seven emergency medicine physicians, two family medicine residents, and three medical students.

Overall, the simulator was positively received with all participants indicating they would recommend it to assist in the training of others. Suggestions focused on enhancing the anatomical representations of both the skin and bones to enhance the learner experience. The content and outcomes of this report support the use of this simulator as part of simulation-based medical education.

## Introduction

Vascular access in trauma is both a critical and essential component of emergency medicine and can considerably influence patient outcomes [1,2]. This has long been recognized as the rate-limiting step in pediatric resuscitation efforts due to anatomic and physiologic differences during development into an adult [1]. Typically, vascular access during trauma is achieved by using intravenous therapy peripherally in the veins of arms and hands to deliver medications and fluid replacement rapidly to the circulation. Unfortunately, peripheral access in children is more difficult to achieve than in adults [2]. In emergencies involving cardiac arrest and shock, peripheral access in pediatric patients is difficult due to extensive peripheral vasoconstriction [3]. This is a considerable concern, given that any delay in establishing vascular access may delay medical interventions, possibly compromising the outcomes of the patient [4].

An alternate route to achieving vascular access is via intraosseous (IO) access. The American Heart Association and the European Resuscitation Council encourage the use of IO procedures if peripheral access is not immediately possible [5,6] because the medullary spaces in bones can act as a “non-collapsible vein,” allowing the rapid and efficacious administration of medications and fluids [3]. The proximal tibia is often used as the primary site of IO injections because it has a large medullary space and can be identified using landmarks [5]. However, despite its effectiveness, IO access appears to remain underutilized in emergency departments (EDs) [7]. Further, in a study examining procedural experience and confidence among graduating medical students, 95% of respondents had never performed an IO procedure [8]. Thus, it has been recommended that continuous medical education programs for medical professionals be implemented to increase the use of IO access [7].

Ota et al. evaluated which IO model best simulated the real procedure and found great variability in preference for either a chicken, turkey, or plastic bone mode, with neither being preferred over the other [9]. More recently, simulated mannequins have been employed for teaching IO injections. Although much more anatomically accurate, these mannequin models are costlier, often costing upwards of 10,000 USD [3]. Therefore, the development of a cost-effective yet anatomically accurate model for IO access training is warranted.

A rapidly evolving educational tool in medicine is the use of three-dimensional (3D) printing to generate 3D haptic models to assist in teaching a certain topic or procedure through simulation-based medical education (SBME) [10]. The growing interest in this type of SBME is partly due to the cost-effective nature of 3D printing and its anatomical accuracy. For example, Rankin et al. discovered through cost analysis research that printing a 3D model over just one week would save enough money to cover the initial cost of the printer [11]. Models can be printed for as little as $0.50 [12], and as time passes, the cost of using 3D printing is projected to decrease even further [13]. It has also been shown that the use of 3D-printed models is more readily available and better represents anatomical features for learning [14,15]. Finally, the use of 3D haptic models is known to be an important educational asset. For instance, it has been reported by learners that SBME using 3D haptic models helped increase their knowledge base and surgical skill set [16]. Thus, a pediatric IO model developed using 3D printing is a possible solution toward combating the financial and anatomical accuracy barriers of current models.

The work presented in this report can be situated as an initial step of the research process guided by the adapted Medical Research Council (MRC) framework for the development and implementation of complex interventions [17]. Specifically, this report aims to outline the development and initial validation of an inexpensive yet anatomically accurate 3D-printed model for pediatric IO injections. In the future, and following the MRC framework, the goal will be to engage in pilot testing to provide more aspects of validity of the model and perform efficacy evaluations. Finally, by engaging in implementation efforts, we plan to incorporate the model into training for medical students, residents, and/or rural physicians as part of SBME and provide the model to rural teaching sites across Canada.

## Technical report

Model overview

**Figure 1:**
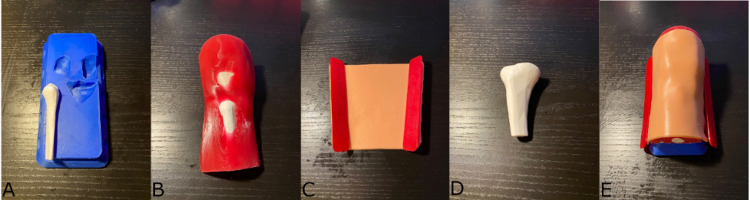
Pediatric IO Infusion Simulator and components. A: base; B: block of muscle and bones; C: tissue with fasteners; D: tibial cartridge; E: assembled simulator IO: intraosseous

The Pediatric IO Infusion Simulator is composed of four components (Figure 1): a base printed in blue polylactic acid (PLA), a block of muscle tissue made from red silicone and bones printed in white PLA, one sheet of replaceable skin molded out of flesh-toned silicone with two skin attachment fasteners printed in red thermoplastic elastomers (TPE), and a tibial cartridge printed in white PLA. All 3D-printed components in this project were created using a Prusa i3 MK3S and an Ultimaker 2+ 3D printer. All silicone molds were made using a mix of Ecoflex™ 00-30 (1:1 A:B ratio).

Two anatomical models of an adult skeleton and external leg were sourced from Creative Commons (Creative Commons, Mountain View, CA, USA). The adult skeleton’s height was 1,706 mm, and the tibia was 380 mm long. The anatomy was scaled down to a pediatric size using the following reasoning: according to a study conducted by Ha et al., the mean tibia length of boys is 223.1 mm and girls 228.5 mm at an age of 36 months [18]. Assuming 230 mm and dividing by 380, this produced a scaling factor of 0.6502; hence, the adult models were scaled by 60%. Blender (Blender Foundation, Amsterdam, Netherlands; www.blender.org), a 3D modeling software, was used to apply a Boolean intersection to the skeleton with a cuboid, a process that combines two or more meshes and preserves the overlapping parts. This allowed extraction of the following portions of the four bones within the knee area: tibia, femur, fibula, and patella. A similar but less complex cuboid was used to produce soft tissue, moving the posterior face anteriorly and causing the bones to protrude slightly. These protruding parts were then subtracted from the base to create recessed shapes, allowing all parts of the model to securely fit in place. Meshmixer (Autodesk Inc., San Rafael, CA, USA) was then used to simplify surfaces and reduce the number of vertices to avoid printing problems related to very fine features. After this process, the four bones were split into separate stereolithography files. A JavaScript program was then written, leveraging an open-source library called OpenJSCAD, allowing further manipulation of the meshes.

Boolean subtraction was used to create the base with recessed shapes for the protruding bones and holes for fasteners that would secure the skin in place. The patella and tibia were lightly glued to the mold bottom using a glue stick, allowing them to be directly embedded within the silicone. Ecoflex™ 00-30 silicone was injected into the clamped mold using 60 mL syringes through two matching injection ports to reduce the risk of air bubbles, providing the muscle tissue, which was molded and then verified to be compatible with the 3D-printed bones and base. A fastener design was generated in OpenJSCAD and several iterations were printed in TPE to provide flexibility and durability. Several iterations of the skin molds were generated in OpenJSCAD, forming a trapezoidal shape with five holes along the side edges. These were then tested with the assembled simulation to determine the optimal skin tension and geometry, readying it for validation.

Each simulator required 549 g of PLA, 19 of TPE, and 835 g of silicone, including the molds and the skin tray. The total material cost was roughly 77 CAD per trainer. A tibial replacement cartridge was designed so that each simulator can be reused after training. This cartridge uses only 23 g of PLA and costs 3.04 CAD of material per unit.

Quality assurance evaluation methods

Feedback on the Pediatric IO Infusion Simulator was collected over a three-month period from December 2020 to February 2021. Due to the coronavirus disease 2019 (COVID-19) pandemic, traditional training “workshops” could not be held as gatherings were prohibited. Instead, an email with instructions was sent out to The Moncton Hospital (TMH) ED staff, residents, and medical students. An evaluation station was made in the ED whereby both simulators and necessary equipment were available so that staff, residents, and medical students could test the simulator at their leisure and complete the evaluation while maintaining social distancing. The simulator was examined periodically to replace used tibial cartridges, collect surveys, and sanitize equipment. In total, seven ED physicians, two family medicine (FM) residents, and three medical students participated in the study.

Testing equipment, procedure, and data collection

**Figure 2:**
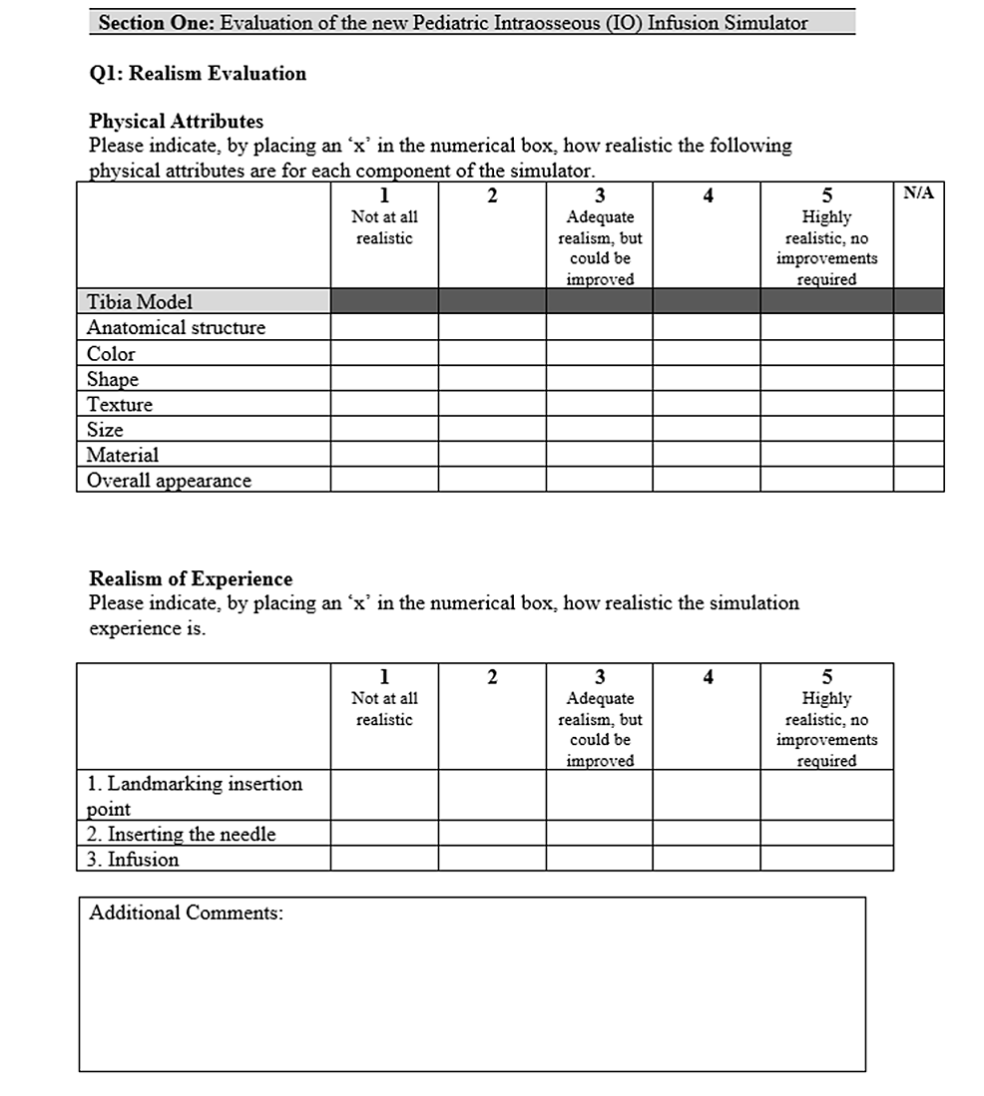
Pediatric IO Infusion Simulator rapid product evaluation survey section I. IO: intraosseous

**Figure 3:**
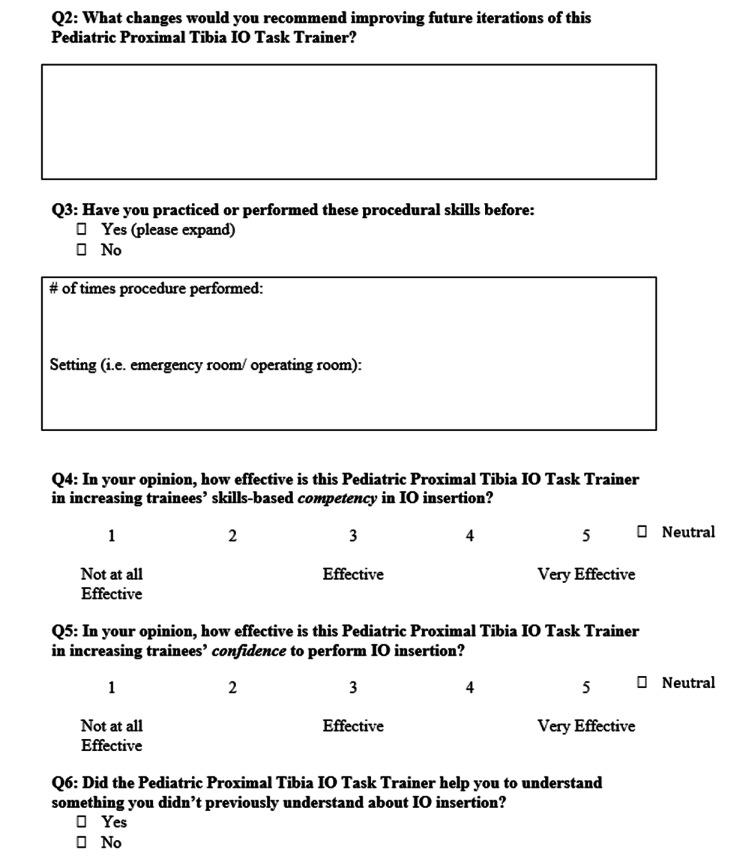
Pediatric IO Infusion Simulator rapid product evaluation survey section II. IO: intraosseous

**Figure 4:**
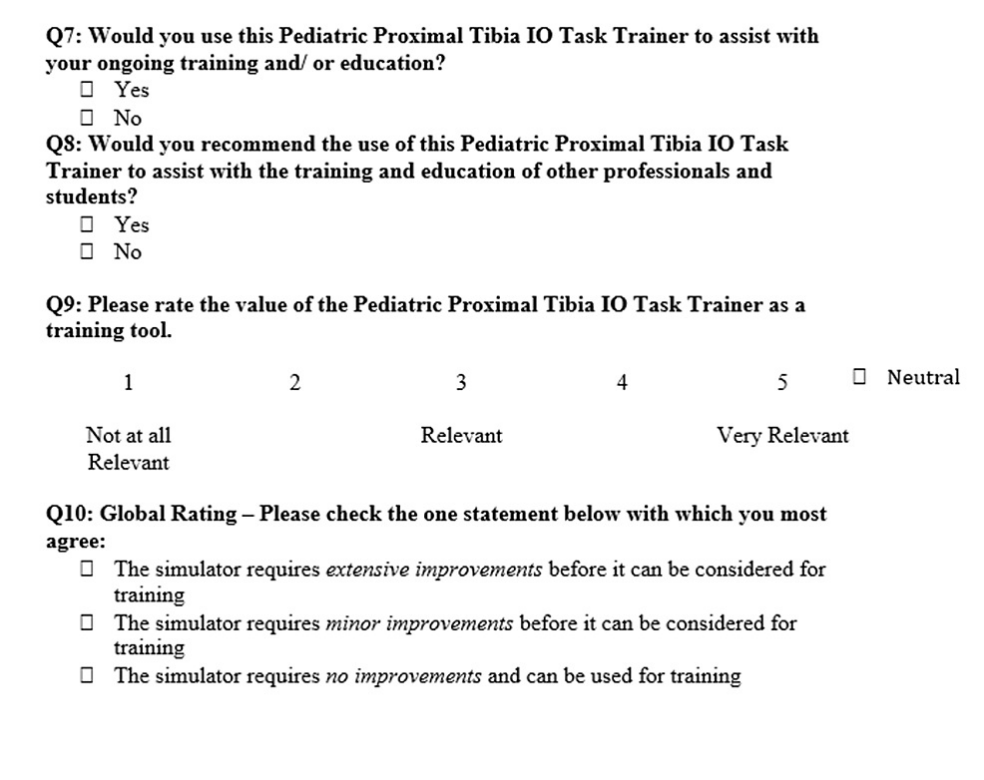
Pediatric IO Infusion Simulator rapid product evaluation survey section III. IO: intraosseous

Due to COVID-19, instructions were provided through email instead of an in-person demonstration. Participants were provided with an Arrow® EZ-IO® Power Driver (Teleflex Medical ResearchTriangle Park, NC, USA), EZ-IO® Needle Set, and the Pediatric IO Infusion Simulator. Participants could practice the procedure as many times as they wished. After testing the simulator, participants were asked to complete a 10-question rapid product evaluation survey (Figures 2-4). The responses from the surveys were then analyzed using Excel for Mac (Microsoft, Version 16.36).

Quality assurance evaluation results

**Figure 5:**
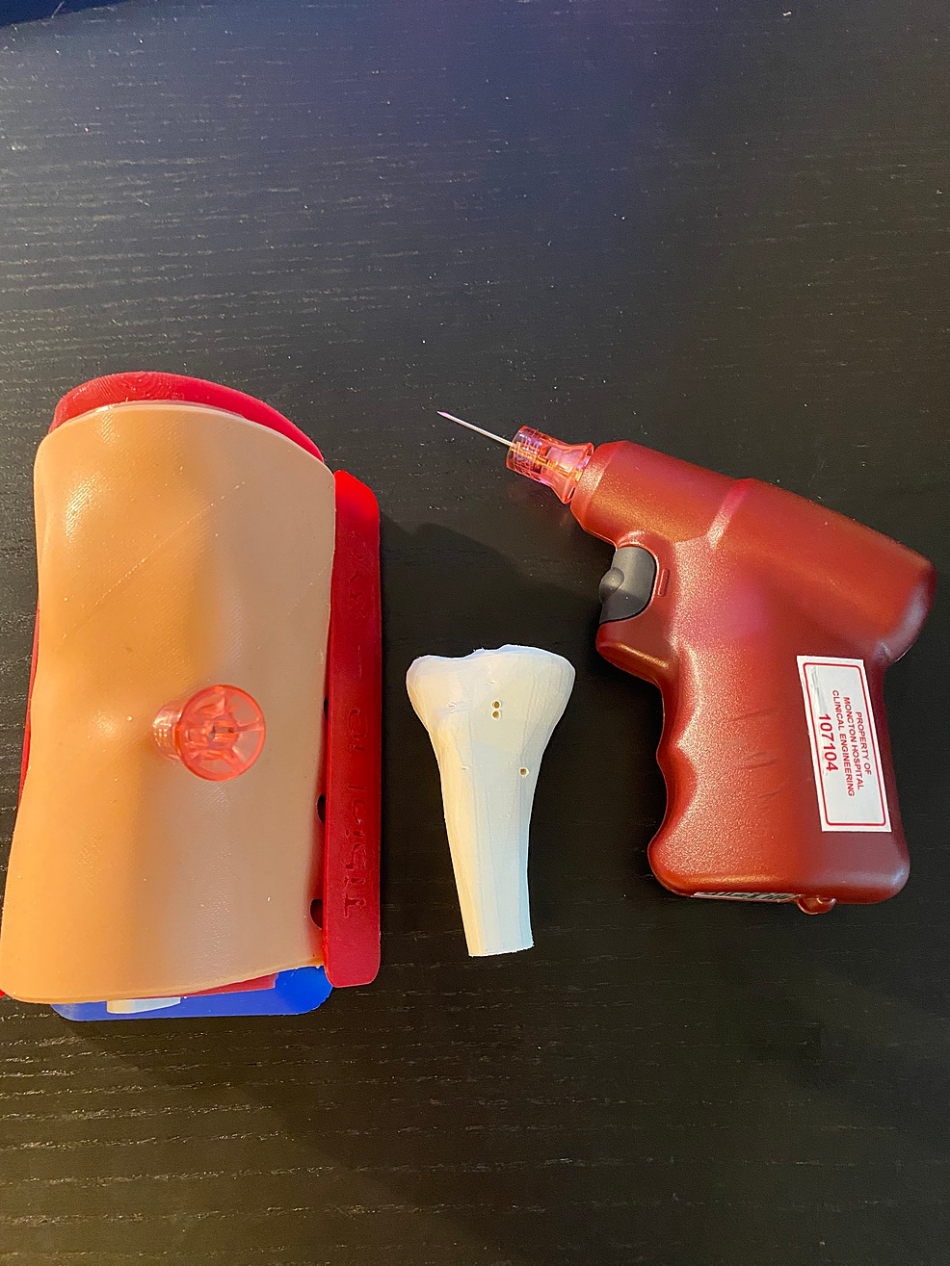
Pediatric IO Infusion Simulator post-testing. IO: intraosseous

Two Pediatric IO Infusion Simulators were provided for testing. Both the replaceable tibial cartridges and skin folds showed little wear during the duration of the testing period (Figure 5). A total of 12 medical professionals participated in the testing process, and all 12 completed the rapid product evaluation survey.

Survey

Question one sought to establish how anatomically accurate the trainer looked and felt compared to the real procedure. A five-point Likert-type scale was used (Figure 2), with a score of 1 indicating not representing anatomical features at all and a score of 5 representing highly anatomically accurate features.

**Table 1:** Question one: physical attributes mean scores. FM: family medicine

Question 1: Physical attributes mean scores	Physicians (N = 7)	FM residents (N = 2)	MD students (N = 3)
Anatomical structure	3.9	4.5	4.3
Color	4.3	4.5	3.3
Shape	4.0	5.0	4.7
Texture	3.7	5.0	3.7
Size	4.0	5.0	5.0
Material	4.1	4.5	4.0
Overall appearance	4.3	5.0	4.0
Landmarking insertion point	4.1	4.0	4.3
Inserting the needle	4.0	3.5	4.3

Question two pursued subjective feedback to improve future iterations of the model. Over half of the responses critiqued the skin material: “skin spins under torque,” “artificial skin not fixed, caught on IO drill with insertion,” “improved superficial skin materials,” “more realistic skin texture, it’s too thick,” “skin rolls around the needle at the end of the insertion,” and “skin replica too elastic, kept getting caught in IO before complete insertion.” Most of the remaining comments centered around the bones: “patella less prominent than expected,” “I mistakenly prodded patella as tibial tuberosity for several rounds,” “bone replica too easy to go through,” and “tibia: flatten anterior aspect of the tibia, too sharp an angle from tibial tubercle.”

Question three assessed participants’ previous experience performing the IO procedure. All (100%) physicians had performed an IO injection at least once before, whereas the majority (80%) of residents and students had not.

**Figure 6:**
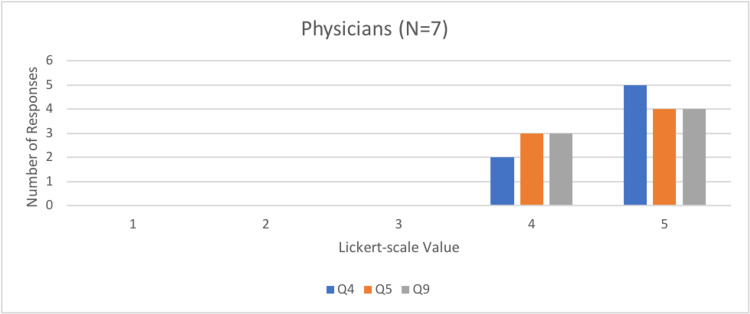
Number of physician responses to questions four, five, and nine of the rapid product evaluation survey.

**Figure 7:**
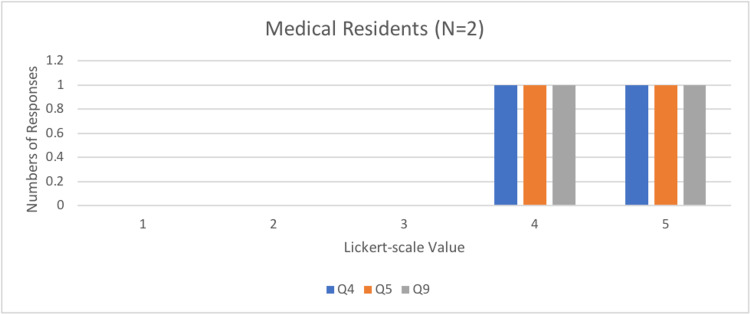
Number of resident responses to questions four, five, and nine of the rapid product evaluation survey.

**Figure 8:**
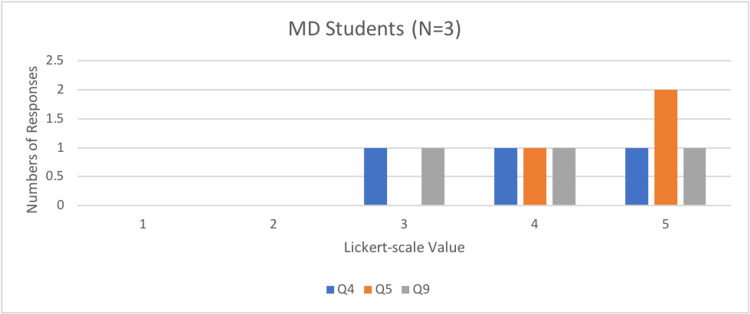
Number of medical student responses to questions four, five, and nine of the rapid product evaluation survey.

Questions four, five, and nine determined how effective and valuable the simulator was in an SBME setting, with a Likert scale of 1 representing not at all effective and 5 representing very effective. Interestingly, most physicians and FM residents found the simulator to be very effective (5/5 on the Likert scale), whereas medical students’ scores ranged from effective (3/5 on Likert-scale) to very effective (Figures 6-8).

**Figure 9:**
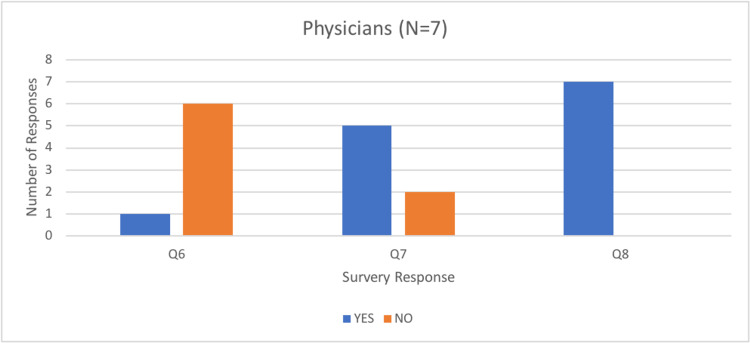
Number of physician responses to questions six, seven, and eight of the rapid product evaluation survey.

Questions six, seven, and eight evaluated whether participants felt the simulator taught them something new and if they deemed it fit as an educational tool for themselves and other learners. Most physicians denied learning anything new about the procedure (86%); however, most residents and medical students (80%) felt they learned something new about the procedure (Figure 9).

**Figure 10:**
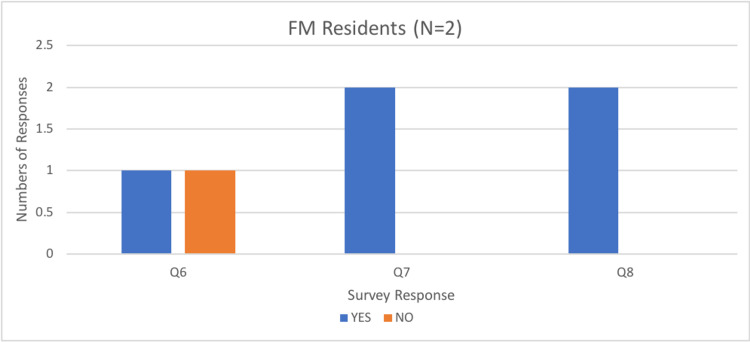
Number of resident responses to questions six, seven, and eight of the rapid product evaluation survey.

**Figure 11:**
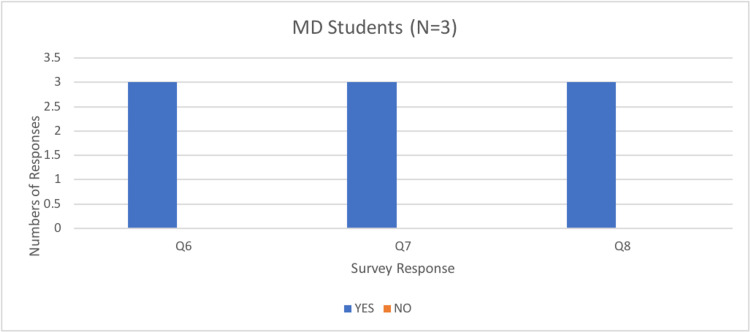
Number of medical students responses to questions six, seven, and eight of the rapid product evaluation survey.

Most participants (92%) responded that they would use the current simulator to assist in their ongoing education and training, and all participants (100%) indicated they would recommend the simulator to assist with the training of others (Figures 9-11).

**Figure 12:**
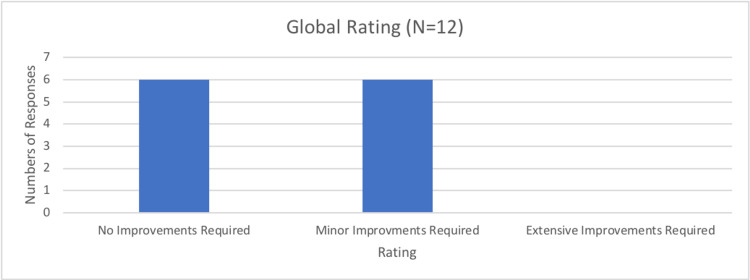
Number of all responses to question 10 of the rapid product evaluation survey.

Finally, question 10 asked participants to judge how much improvement they thought the simulator required before being validated as a training tool, with none of the respondents indicating extensive improvements were required. However, 50% of respondents indicated that some minor improvements were required (Figure 12).

## Discussion

This report aimed to outline the development and initial validation of an inexpensive yet anatomically accurate 3D-printed model for pediatric IO injections. Overall, the Pediatric IO Infusion simulator was well-received and met most expectations as an educational training tool by medical students, FM residents, and ED physicians. As shown in the results, 100% of the participants indicated that they would recommend the simulator to assist in the training of others. However, some improvements to enhance the representation of anatomical features were suggested, most of which centered around the skin material and the anatomy of the bones. This is supported quantitatively with the lowest mean Likert scale scores in “texture,” “anatomical structure,” and “landmarking insertion point” and qualitatively as described above. Some improvements may be required for future iterations of the trainer to enhance the learner experience. Some of the improvements, based on the feedback above, should include improving the texture of the skin material and tibia, better anatomical accuracy of the tibial tuberosity so that it is not mistaken for the patella, and enhancing the skin attachment so it does not twist under the torque of the IO drill.

One should consider the fact the trainer costs 77 CAD to be produced whereas a comparable “infant I.O. infusion simulator” by GTSimulators (GTSimulators, Florida, USA) costs 795.95 USD, which is 10 times higher [19]. Additionally, the Pediatric IO Infusion Simulator can be accessed and produced in any location with access to a 3D printer. This is especially useful for medical training programs that place students in rural hospitals with limited access to training materials. Although a pediatric IO procedure is rare, it can be critical in resuscitative efforts. Hence, having prior knowledge/experience can build learner confidence before having to perform it in a real-life scenario.

Finally, it is important to note the limitations of this study and how they may have impacted findings. For instance, the relatively low number of participants impacts the validity of the results. Ideally, a much larger sample size from various urban and rural sites would be needed to enhance the validity of the model. Additionally, due to the COVID-19 pandemic, the study had to be conducted remotely, with participants being emailed the instructions, as opposed to the original plan of holding a “workshop.” Unfortunately, this prevented an in-person initial demonstration of the technique. Finally, “content experts” were limited to one hospital. Ideally, a larger and more diverse cohort of individuals would have been obtained.

## Conclusions

The purpose of this report was to describe the development and subsequent evaluation of a 3D-printed Pediatric IO Infusion Simulation Model. The study sought to assess the utility of this high-fidelity, low-cost simulation model. Most of the findings indicated that the trainer is a useful educational tool that can be helpful to medical learners. Suggestions focused on enhancing the anatomical representations of both the skin and bones to enhance the learner experience. Hopefully, after implementing the suggestions from content experts to enhance the validity of the trainer, it can be distributed with the intent of providing a training resource for both medical learners and professionals in practice.
